# Overview of Graves Ophthalmopathy Literature From 1999 to 2019: Bibliometric Analysis

**DOI:** 10.2196/24831

**Published:** 2021-09-28

**Authors:** Jiamin Cao, Nuo Wang, Shiying Hou, Xin Qi, Yu Chen, Wei Xiong

**Affiliations:** 1 Third Xiangya Hospital Central South University Changsha China; 2 Second Xiangya Hospital Central South University Changsha China

**Keywords:** Graves ophthalmopathy, bibliometric analysis, CiteSpace, Web of Science

## Abstract

**Background:**

Research on Graves ophthalmopathy has increased remarkably over the last 2 decades; however, few statistical analyses of the data presented in these publications have been conducted.

**Objective:**

This study aims to detect and analyze emerging trends and collaboration networks in Graves ophthalmopathy research.

**Methods:**

Graves ophthalmopathy–related publications from 1999 to 2019 were collected from the Web of Science Core Collection Database. Collected publications were restricted by category (article or review) and language (English). Bibliometric analyses included changes in the annual numbers of publications, journals, authors, countries, institutions, keywords, and references.

**Results:**

In total, 3051 publications that met the criteria were collected. The number of annual publications has exhibited an increasing trend over the last 20 years. The journal *Thyroid* ranked first, publishing 183 Graves ophthalmopathy–related studies. There was no evidence of a relationship between impact factor (IF) and the number of publications (*P*=.69). The author Smith TJ had the largest number of publications on Graves ophthalmopathy (n=83). Of the countries that had published Graves ophthalmopathy–related articles, the United States had the largest number (n=784) and the highest centrality (0.18). Among institutions, the University of Pisa (Italy) contributed the most Graves ophthalmopathy–related articles (n=114). The most recent burst keywords (proliferation, rituximab, and selenium) and references may provide clues on emerging trends in research and clinical practice.

**Conclusions:**

This bibliometric analysis highlights countries, institutions, and authors who contributed to Graves ophthalmopathy–related publications. Emerging trends in Graves ophthalmopathy research, based on burst keywords and references, may provide clues relevant to clinical practice and future research.

## Introduction

### Graves Ophthalmopathy

Graves ophthalmopathy is an organ-specific autoimmune disease related to Graves disease (GD), which is among the most frequent extrathyroid manifestations of this condition [1]. The most common clinical features of Graves ophthalmopathy are eyelid retraction and exophthalmos, with incidence rates of 90% and 62%, respectively [2]. Other manifestations of Graves ophthalmopathy include restrictive extraocular myopathy, exposure keratitis, and optic nerve dysfunction. The pathogenesis of Graves ophthalmopathy is unclear, with numerous issues yet to be resolved. Recently, orbital fibroblasts have been proposed to play an important role in adipogenesis and fibrosis. In vivo, orbital fibroblasts differentiate into adipocytes and myofibroblasts in response to peroxisome proliferator-activated receptor-γ and transforming growth factor β, respectively, and these are the main pathological changes in the inactive stage of Graves ophthalmopathy [3]. Recommended first-line therapeutic strategies include glucocorticoid and thyroid function control [4]. Many new therapeutic strategies for Graves ophthalmopathy have recently emerged, including teprotumumab, which can reduce the degree of exophthalmos [5]. Given the numerous Graves ophthalmopathy–related publications, it is important to analyze these research studies to provide an overview of the field. Although some previous reports have introduced state-of-the-art bibliometric analysis of related diseases, these publications also included other conditions and did not provide an accurate description of the single disease, Graves ophthalmopathy [6,7]. Furthermore, other bibliometric analyses of eye-related diseases, such as diabetic retinopathy and glaucoma, have been reported; however, bibliometric data related to thyroid-associated ophthalmopathy are lacking [8,9].

### Bibliometric Analyses

Systematic reviews, meta-analyses, and bibliometric analyses can be used to describe developments in a specific field. Systematic reviews and meta-analyses are primarily used to compare the effectiveness or side effects of treatment strategies or drugs and can provide decisive or suggestive data; however, they do not provide descriptive statistical analysis of research in a field [10]. Bibliometric analysis is a statistical analysis approach that can be used to describe the characteristics of large-scale data and determine the main development trends based on the results of database searches on a given topic [11]. Through bibliometric analysis, data on the number of publications, countries, institutions, authors, and research hotspots are extracted, providing visual results describing research status [12]. Bibliometric analysis is a quantitative and visual process that includes the detection, description, evaluation, and monitoring of published research studies [13]. CiteSpace 5.6R5 (32-bit) is a bibliometric analysis tool developed by Chaomei Chen in 2004 and has been used by many researchers [13]. For example, CiteSpace has been used to analyze the development and trends in research on valvular heart disease [14]. In our study, CiteSpace was the main bibliometric analysis tool used.

### Destination

In this research, we aim to analyze the characteristics of Graves ophthalmopathy research and assess the tendencies and perspectives related to Graves ophthalmopathy over the past 20 years, from 1999 to 2019.

## Methods

### Data Sources and Search Strategies

The Web of Science database is a collection of multidisciplinary academic journals and an authoritative citation information source that serves as a primary scientific database for many researchers [15]. The data source for this study was the Web of Science Core Collection (WoSCC) database, which includes the following sources: Science Citation Index Expanded, Social Sciences Citation Index, Arts & Humanities Citation Index, Conference Proceedings Citation Index-Science, Conference Proceedings Citation Index-Social Science & Humanities, Emerging Sources Citation Index, and Current Chemical Reactions [15]. The research strategies were topic search, that is, *Graves ophthalmopathy* OR *Graves orbitopathy* OR *thyroid-associated ophthalmopathy* OR *thyroid eye disease*, over the period 1999-2019. On July 21, 2020, a total of 3848 records were obtained. After restricting the document type to article and review and language to English, 3051 records remained (Multimedia Appendix 1).

### Data Collection and Preprocessing

All records and references were saved with a name in the format download_**.txt, as this format is recognized by CiteSpace [16]. Data from publications collected from WoSCC were saved in documents, including title, authors, countries, institutions, abstracts, keywords, journal, and publication date. After the removal of duplicates using CiteSpace, records were classified by year. As the same author name could be presented in two or three forms (eg, *TJ Smith* or *Terry J Smith*), we replaced different forms found with 1 form in the raw data before detecting the cooperating relationships among authors. Country and institution names were not preprocessed, as they had only single names in the raw data.

### Bibliometric Analysis

We used CiteSpace 5.6R5 (32-bit). Preprocessed data were uploaded to CiteSpace, and bibliometric analysis was performed according to the information included in the data documents [13]. Network maps visualizing collaborations among journals, authors, countries, and institutions were generated. In network maps, points represent an author, country, or institution, and lines represent relationships between points. Larger points and stronger lines represent more records and stronger collaboration relationships, respectively [11]. Burst detection analysis of keywords and references was used to discern trends, outlooks, and research interests [17]. Time slicing was conducted from January 1999 to December 2019, with 1 year per slice. Default selection criteria parameters were used, including the *pruning* parameters, *pathfinder* and *pruning sliced networks*, and *visualization* parameters, *cluster view-static*, and *show merged network*.

Centrality, also referred to as betweenness centrality, is an index that can be used to illustrate the importance of a node in a network [13]. In CiteSpace, centrality can be used to identify turning points and measure the co-operation and participation of countries in literature production. Higher centrality values indicate closer co-operation relationships of a country with others, a higher degree of participation, and a greater impact if deleted [18]. Centrality was calculated using the following equation:

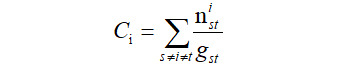



where g_st_ represents the number of shortest paths from node s to node t and n^i^_st_ represents the number of shortest paths that pass through node i in g_st_.

GraphPad Prism 8 and web-based bibliometric analysis platforms were used to analyze annual publication outputs and count numbers of publications according to journal. Oracle Crystal Ball (11.1.2.4.400) was used to predict the trend in the numbers of publications. Furthermore, Hirsch index (H-index) values were searched from the Web of Science; H-index values indicate that researchers have published h papers and that each paper was cited at least h times and can be used to describe the cumulative impact of an author, country, or institution [19]. Journal IFs were obtained from the 2019 version of Journal Citation Reports, which are used as a measure of the scientific value of research [20]. IBM SPSS Statistics 24 was used to create fitting curves for the number of publications and analyze relationships between IF and numbers of Graves ophthalmopathy–related publications in journals. SPSS 24 was also used for hypothesis testing.

## Results

### Publication Outputs

**Figure 1:**
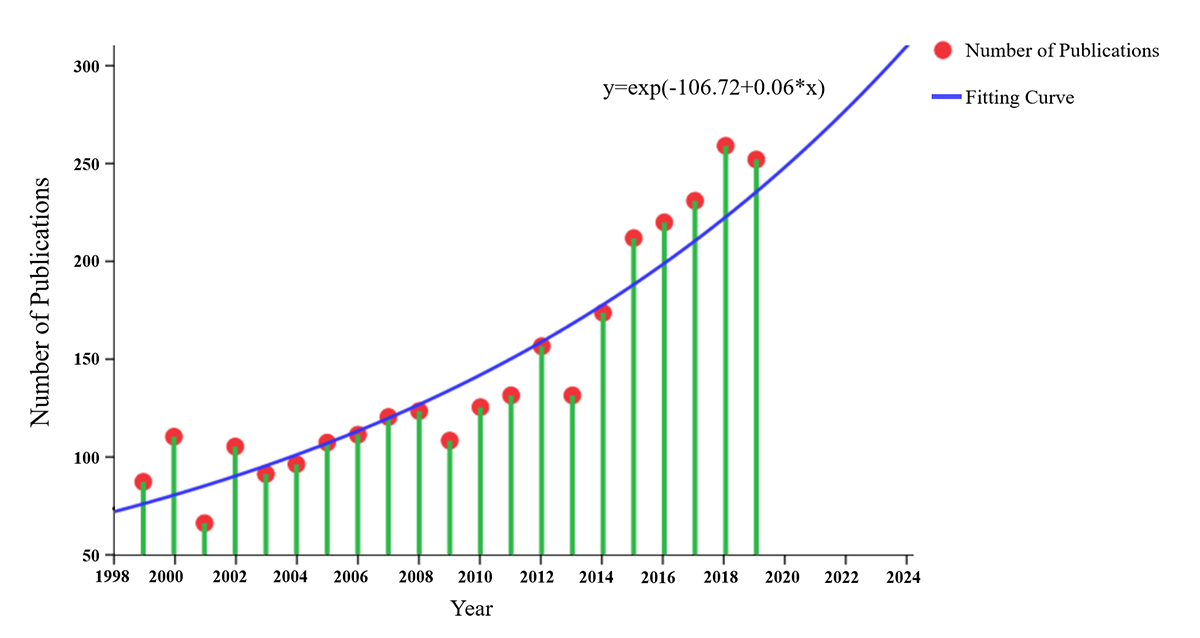
Number of Graves ophthalmology–related publications from 1999 to 2019.

Searching the Web of Science database using the search conditions described earlier yielded 3051 records that met the criteria that were included in further analysis. The distribution of Graves ophthalmopathy–related publication numbers annually from 1999 to 2019 is shown in Figure 1. The total number of publications increased from 89 in 1999 to 253 in 2019. The largest number of Graves ophthalmopathy–related publications over the past 20 years (1999–2019) occurred in 2018 (n=260). The number of publication outputs decreased in 2001, 2003, 2009, and 2013. An exponential fitting curve was generated using SPSS 24 (*R*^2^=0.86; *P*<.001; Figure 1), which showed a significant increase in the number of publications over the last two decades. Furthermore, according to the exponential fitting curve, the number of outputs is predicted to continue increasing over the next 5 years, and the probability of increase is 92.8% according to the Oracle Crystal Ball.

### Distribution of Graves Ophthalmopathy–Related Publications in Journals

Information about journals from the Web of Science was analyzed to determine rank information. The 3051 identified publications were published in 691 journals. The top 10 journals with the highest number of publications are listed in Table 1. The total number of publications in these 10 journals was 929, which accounted for 30.44% (929/3051) of all publications. The number of publications in the top 10 journals ranged from 50 to 183. The journal *Thyroid* contributed the highest proportion of publications (n=183) among all the journals included in this research, followed by *Ophthalmic Plastic and Reconstructive Surgery* (n=153) and *Journal of Clinical Endocrinology & Metabolism* (n=136). Furthermore, the *Journal of Clinical Endocrinology & Metabolism* had the highest total and mean number of citations. The 2019 IF of the top 10 journals ranged from 1.113 to 8.470, and 8 journals had an IF >3 and published 828 (27.1%) of the 3051 Graves ophthalmopathy–related papers from 1999 to 2019. The relationship between IF and the number of publications in the top 10 journals was assessed by Pearson correlation analysis in SPSS.22, which revealed no significant correlation (*R*^2^=–0.145; *P*=.69). These data demonstrate that publishing papers in journals with a high IF remains challenging.

**Table 1:** Top 10 journals with the most published articles.

Rank	Journal	Impact factor (2019)	Publications, n (%)	Total number of citations	Mean number of citations
1	Thyroid	5.227	183 (6)	2500	13.66
2	Ophthalmic Plastic and Reconstructive Surgery	1.133	153 (5.01)	956	6.25
3	Journal of Clinical Endocrinology & Metabolism	5.339	136 (4.46)	3995	29.38
4	European Journal of Endocrinology	5.308	77 (2.52)	1552	20.16
5	Clinical Endocrinology	3.380	76 (2.49)	1402	18.45
6	Journal of Endocrinological Investigation	3.397	75 (2.46)	859	11.45
7	Eye	2.455	68 (2.22)	511	7.51
8	Investigative Ophthalmology & Visual Science	3.470	60 (1.97)	619	10.32
9	British Journal of Ophthalmology	3.611	51 (1.67)	761	14.92
10	Ophthalmology	8.470	50 (1.61)	796	15.92

### Distribution by Author

**Figure 2:**
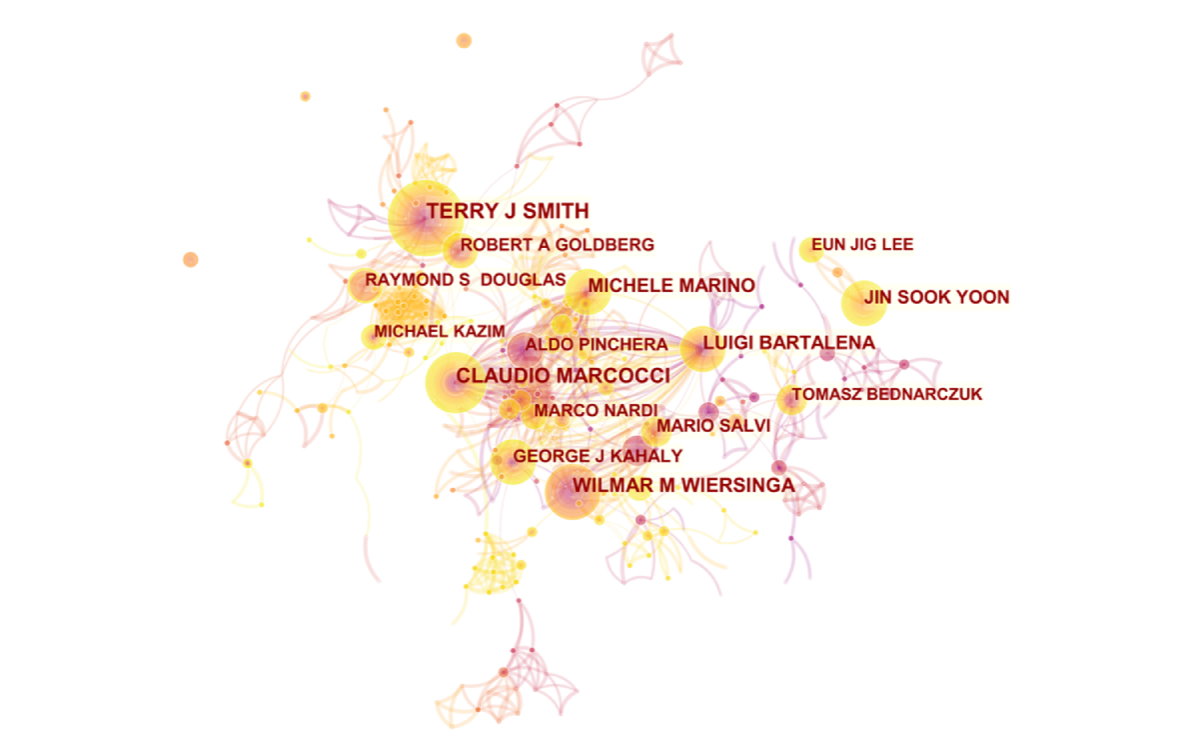
Network of co-operation relationships among authors generated using CiteSpace.

A total of 9660 authors contributed to the studies included in this research. Co-operation relationships among authors were analyzed using the coauthor tool in CiteSpace. Co-operation among authors was visualized as a network map to illustrate potential partnerships (Figure 2). Co-operation relationships are represented by connections between nodes, with thicker connections indicating closer co-operation. The node size represents the number of author outputs, with larger size indicating more outputs. The top 10 authors with the largest number of Graves ophthalmopathy research publication outputs are presented in Table 2, and the data came directly from the Web of Science on August 4, 2020. Among the top 10 authors, Smith TJ had the largest number of articles (83/3051, 2.72%), followed by Marcocci C (73/3051, 2.42%) and Kahaly GJ (68/3051, 2.23%). Wiersinga WM (H-index=75) had the highest H-index among the top 10 authors, followed by Bartalena L (H-index=61), Marcocci C (H-index=60), and Hegedus L (H-index=60). The assessment of relationships between author and H-index by Spearman rank correlation in SPSS 24 detected no significant relationships (*P*=.38), possibly indicating that the number of author outputs is not an important factor influencing the H-index.

**Table 2:** Top 10 authors of the most articles.

Rank	Author	Publications	Percentage of 3051	H-index
1	Terry J Smith	83	2.72	54
2	Claudio Marcocci	73	2.4	60
3	George J Kahaly	68	2.2	46
4	Wilmar M Wiersinga	62	2.0	75
5	MicheleI Marino	55	1.8	26
6	Luigi Bartalena	53	1.7	61
7	Jin Sook Yoon	46	1.5	20
8	Mario Salvi	42	1.4	33
9	Raymond S Douglas	40	1.3	24
10	Laszlo Hegedus	39	1.3	60

### Distribution by Country

**Figure 3:**
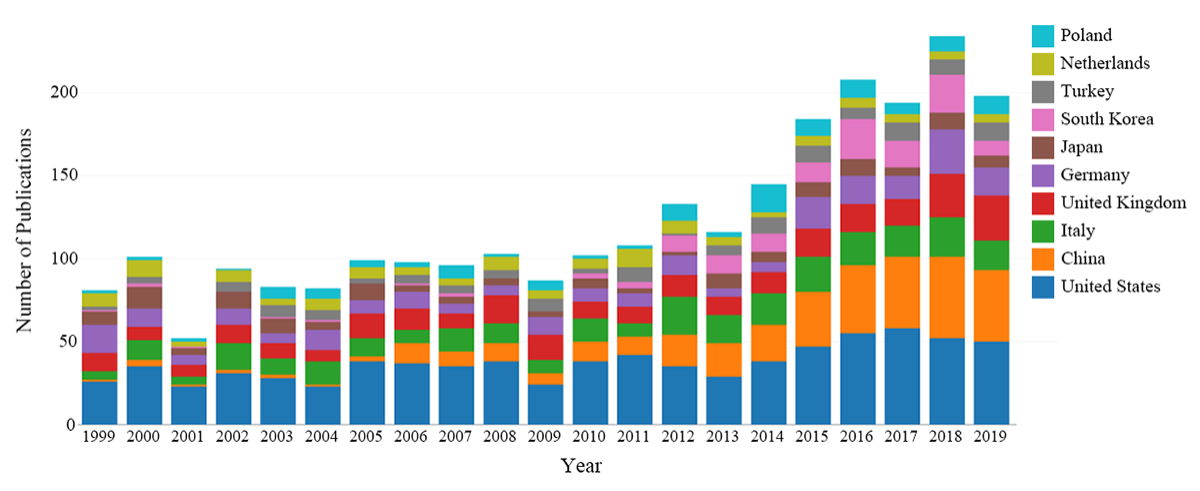
Annual publications distributed according to country.

**Figure 4:**
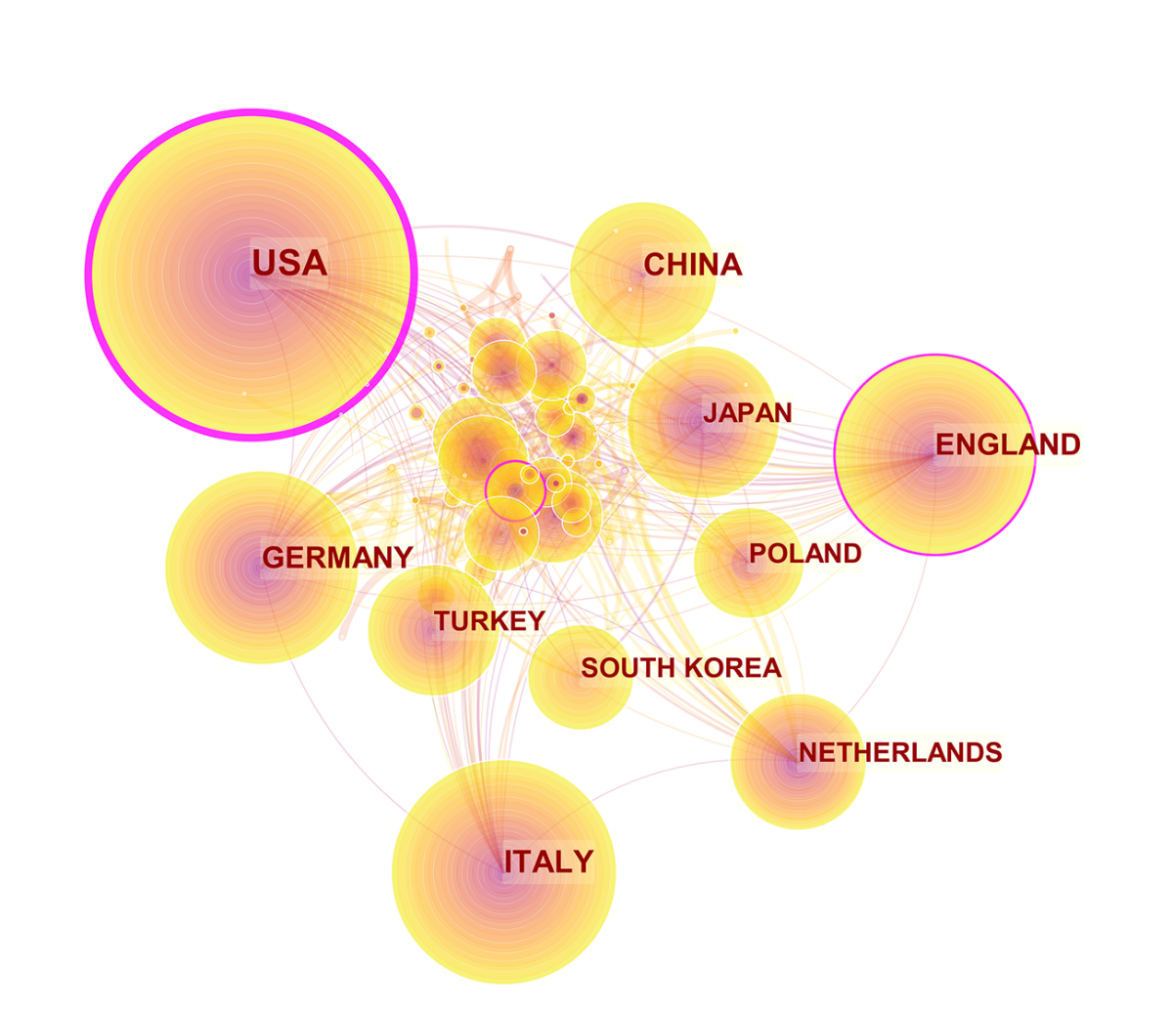
Network of co-operation among countries generated using the CiteSpace web-based analysis platform.

The 3051 outputs included in this study originated from 83 countries or regions. In data preprocessing, we included data from Taiwan (78 publications) into those from China. The number of countries involved in the outputs and their distribution were analyzed using web-based bibliometric analysis platforms, and the results are shown in Figure 3. Different colors represent different countries, and the length of bars indicates the number of articles, where longer bars indicate a higher number of publications. The co-operation relationships are shown in Figure 4. Circles represent centrality, with wider circles representing higher centrality. Connections between nodes represent co-operation between the countries. Detailed publication information for the top 10 countries is presented in Table 3. Among the top 10 countries, the United States had the most publications (784/3051, 25.69%), followed by China (350/3051, 11.47%) and Italy (301/3051, 9.87%). Furthermore, the United States had the largest centrality (0.18), followed by England (0.15) and Turkey (0.09), demonstrating that the United States had the highest level of co-operation with other countries. Moreover, country centrality was not significantly associated with the number of outputs from the country (Spearman rank correlation, *P*=.59).

**Table 3:** Top 10 countries with the most articles.

Rank	Country	Publications	Percentage of 3051	Centrality
1	United States	784	25.7	0.18
2	China	350	11.5	0.00
3	Italy	301	9.9	0.00
4	Germany	240	7.9	0.00
5	England	235	7.7	0.15
6	Japan	141	4.6	0.04
7	South Korea	134	4.4	0.04
8	Turkey	129	4.2	0.09
9	Netherlands	128	4.2	0.07
10	Poland	127	4.2	0.05

### Distribution by Institution

**Figure 5:**
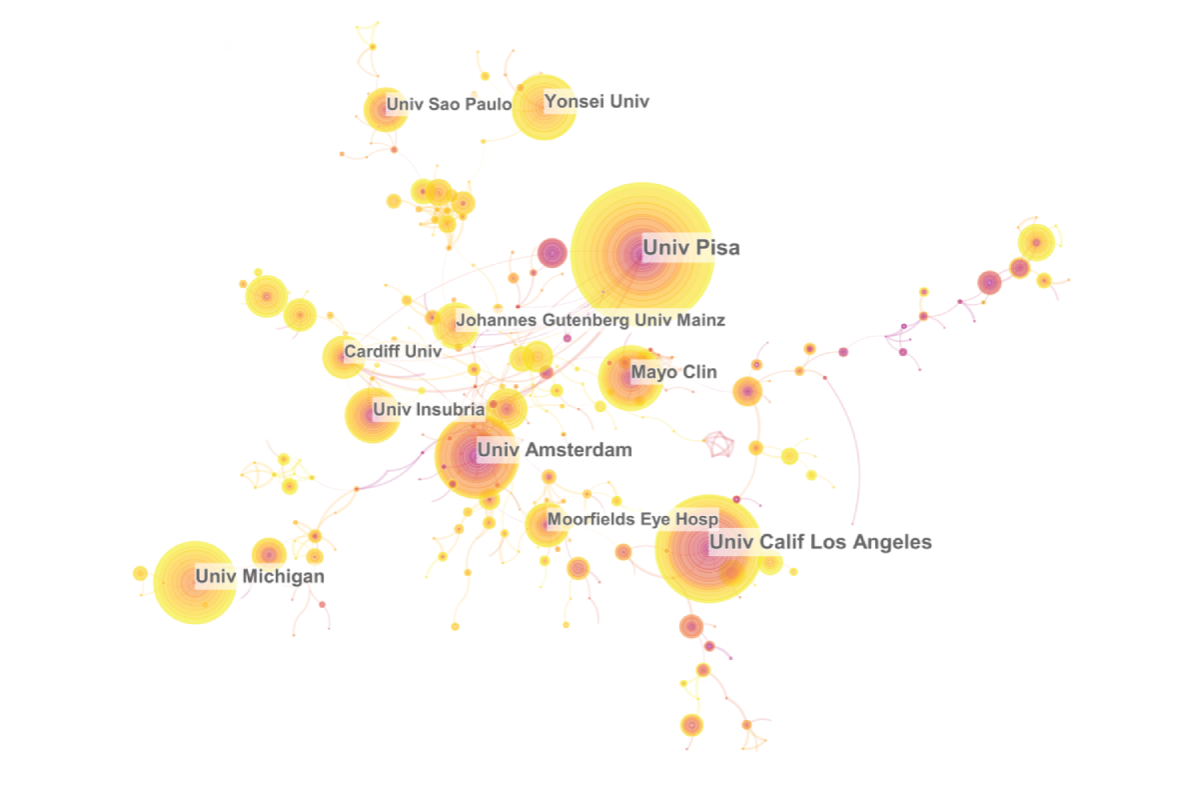
Institution co-operation network.

A total of 2620 institutions contributed to 3051 publications on Graves ophthalmopathy research. The co-operation relationships among institutions are shown in Figure 5, and the top 10 institutions according to the number of publication outputs are presented in Table 4. Among the top 10 institutions, the University of Pisa ranked first with 114 publications, followed by the University of California, Los Angeles (n=91), and the University of Amsterdam (n=72). Of the top 10 institutions, 3 were in the United States and 2 in Italy, suggesting that these organizations made outstanding contributions to the field. Interestingly, the number of institutions in a country was proportional to the number of literature outputs (Spearman rank correlation, *r*=0.77; *P*=.005), demonstrating that the achievements of countries could not be attributed to specific institutions and that more institutions should be constructed to contribute to progress in the field of Graves ophthalmopathy research.

**Table 4:** Top 10 institutions publishing the most articles.

Rank	Institution	Publications, n	Percentage of 3051	Country
1	University of Pisa	114	3.7	Italy
2	University of California, Los Angeles	91	3.0	United States
3	Universiteit van Amsterdam	72	2.4	Netherlands
4	University of Michigan	69	2.3	United States
5	Mayo Clinic	59	1.9	United States
6	Yonsei University	52	1.7	South Korea
7	University of Insubria	50	1.6	Italy
8	Johannes Gutenberg University Mainz	46	1.5	Germany
9	University of Sao Paulo	45	1.5	Brazil
10	Moorfields Eye Hospital	43	1.4	United Kingdom

### Analysis of Keywords

**Figure 6:**
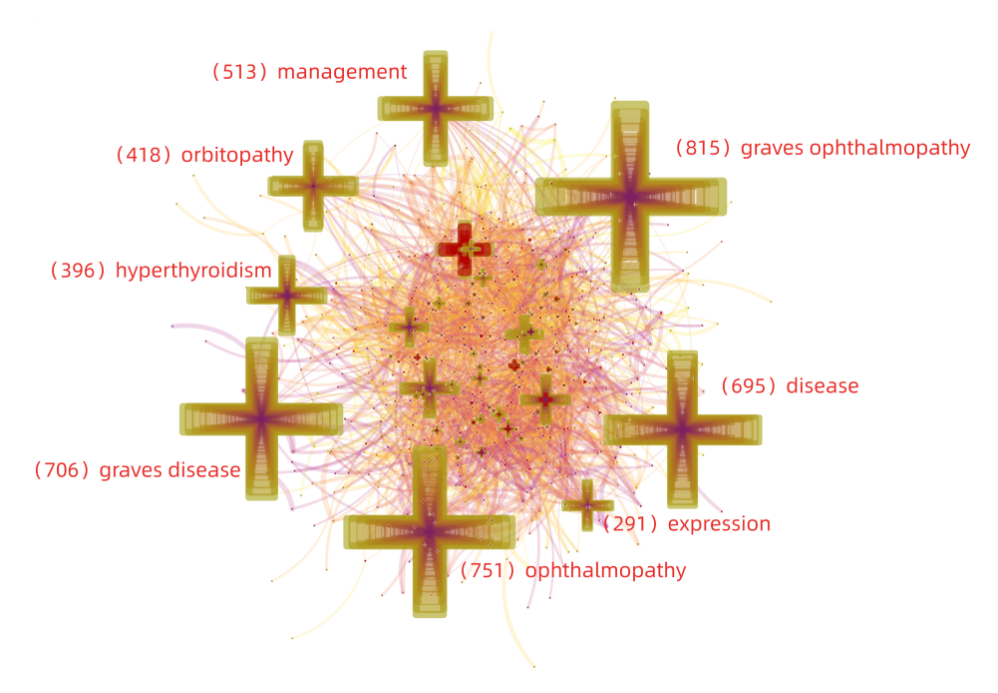
Keywords network.

**Figure 7:**
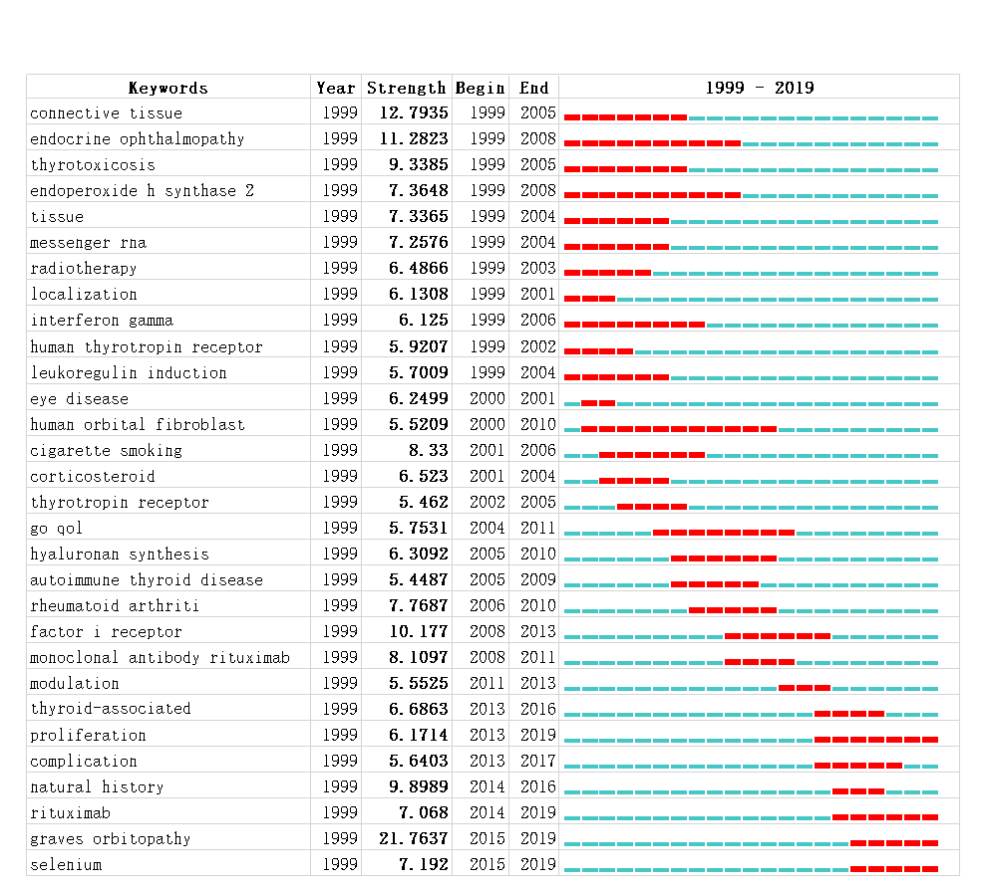
Top 30 strongest burst keywords.

Keyword analysis using CiteSpace revealed a network of keywords, where larger nodes indicated a higher occurrence. As shown in Figure 6, *Graves ophthalmopathy* occurred most frequently (n=815), followed by *ophthalmopathy* (n=751) and *Graves disease* (n=706). Next, keywords were sorted by burst strength; the top 30 keywords with the strongest citation bursts are presented in Figure 7. The occurrence of a burst indicates a sharp increase in keyword occurrence during a specific period and represents hot topics and field dynamics. The analysis of keyword bursts continuing until 2019 could indicate hot topics in the field of Graves ophthalmopathy research. Of the 30 bursts detected, proliferation (2013–2019), rituximab (2014-2019), and selenium (2015-2019) were hot topics in recent years. *Graves orbitopathy* was also a burst keyword; however, it is a theme word and cannot be considered a hot topic.

### Analysis of References

**Figure 8:**
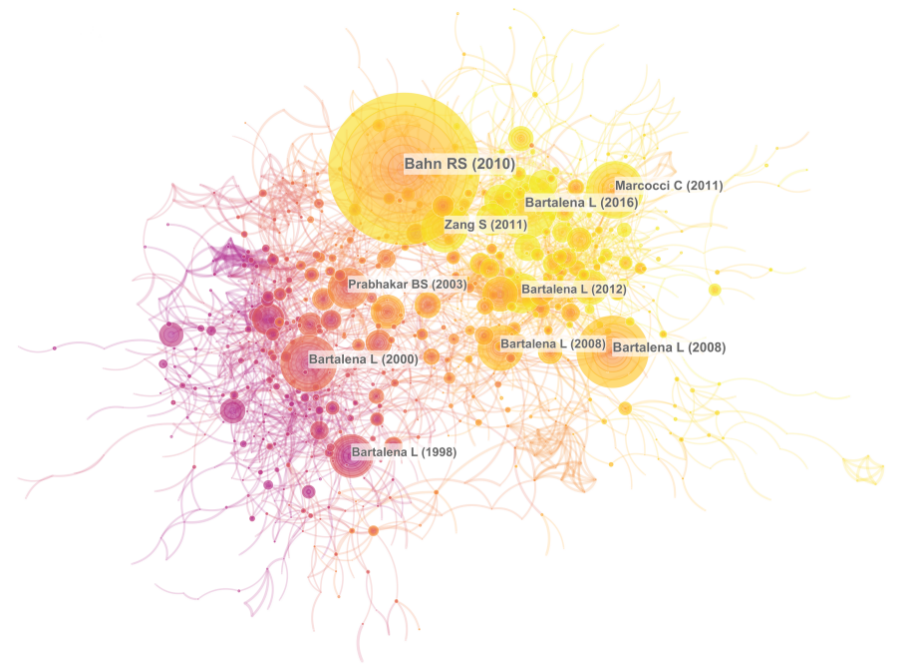
Reference network.

**Figure 9:**
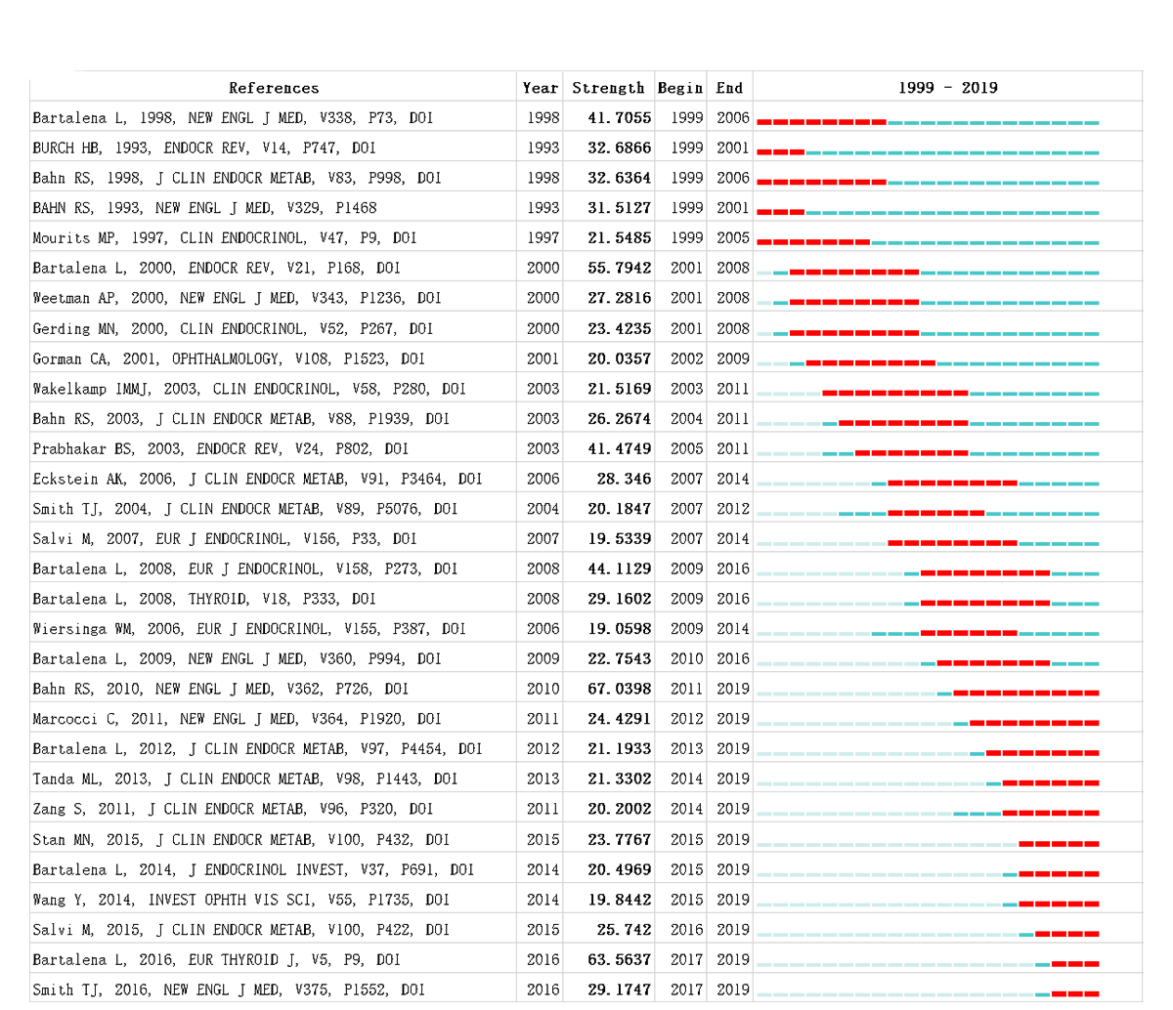
Top 30 strongest references.

Reference analysis is an important part of the bibliometric studies. Reference analysis using CiteSpace generated a reference network, illustrating the relationships among the reference citations. The top 30 strongest reference bursts, which can be considered fundamental knowledge in the field, are presented in Figure 8. Bahn RS (2010) [21] led the citation bursts to 2019, followed by Bartalena et al [4] and Smith et al [1] (Figure 9).

## Discussion

### Principal Findings

In this study, a bibliometric analysis of Graves ophthalmopathy publications from 1999 to 2019 was conducted using CiteSpace, GraphPad Prism 8, SPSS, and web-based bibliometric analysis platforms. Our exponential fitting curve analysis results show that the number of publications continues to increase, indicating that more research and articles will contribute to this field. Hence, the Graves ophthalmopathy field will continue to be a hot topic in the future, both because of the many points requiring clarification and the substantial physical and mental impacts of Graves ophthalmopathy on patients.

Of the top 10 profiled countries, 9 were high-income nations, with China being the only low-income country. The United States had the largest number of Graves ophthalmopathy research outputs (n=784) and the highest centrality (0.18). China had 350 Graves ophthalmopathy research outputs, ranking second in terms of the number of publications. As a transitional country, China has the potential to progress much further in Graves ophthalmopathy research and would benefit from more co-operation with other countries. The top 10 institutions were responsible for a total of 641 publications (20% of 3051), and 8 were colleges and universities in high-income countries, including the United States (n=2), Italy (n=2), the Netherlands (n=1), South Korea (n=1), Germany (n=1), and 1 transitional country, Brazil (n=1). The other 2 institutions, the Mayo Clinic and Moorfields Eye Hospital, were hospitals in the United States and the United Kingdom, respectively. The number of publications may be related to economic prosperity at the country level.

Some excellent authors have been identified in our analysis. Smith TJ had the largest number of Graves ophthalmopathy–related publication outputs (n=83) over the last 20 years; Marcocci C (n=73) ranked second, and Kahaly GJ (n=68) ranked third. The H-index is used to describe the breadth of the impact of an author’s scientific research. Wiersinga WM had the highest H-index (75), despite not having the most Graves ophthalmopathy–related publication outputs over the last 20 years. All these authors devoted themselves to the development of this field. For example, Smith TJ found that patients with Graves ophthalmopathy had higher levels of fibrocytes in the blood than those without Graves ophthalmopathy [22]. Furthermore, Marcocci et al [23] found that patients with Graves ophthalmopathy who undergo total thyroid ablation may have better outcomes and health improvements than those who undergo thyroidectomy alone. Moreover, Kahaly et al [24] reported that teprotumumab resulted in better outcomes than treatment with a placebo.

As shown in Figure 7, burst keywords were detected using CiteSpace. Among the top 30 strongest burst keywords, proliferation, rituximab, and selenium remained burst keywords by the end of 2019, suggesting that they may occur frequently in the coming years and represent emerging trends. These burst keywords were introduced as follows.

### Proliferation

As Graves ophthalmopathy is an autoimmune disease, inflammation of orbital tissue is commonly involved, according to recent research. The proliferation of fibrocytes and fibroblasts is considered an important aspect of the pathogenesis of Graves ophthalmopathy. Many studies have focused on the regulation of fibroblast proliferation, with the aim of determining possible Graves ophthalmopathy therapy approaches. For example, gypenosides promote fibroblast proliferation, inflammation, and fibrosis to regulate Graves ophthalmopathy progression [25]. Furthermore, chitosan inhibits fibroblast proliferation and adipogenesis, which may have therapeutic effects on Graves ophthalmopathy [26]. Although there are many other biological processes, including cell death and degeneration, the inclusion of proliferation as a burst keyword may predict that more research in this field will focus on this cellular process in the future.

### Selenium

Selenium was identified as a component of an enzyme that activates thyroid hormones in the 1990s. Selenium supplementation can regulate thyroid function by decreasing serum T4 concentration, and pregnant women with GD have lower selenium levels than those without GD [27]. Research on the effects of selenium on Graves ophthalmopathy suggests that it may improve disease outcomes by preventing cell damage and decreasing cytotoxic oxidative stress damage to fibroblasts [28]. Indeed, there are many adjuvant therapies available, among which supplementation with selenium is a major method recommended in the 2016 European Thyroid Association/European Group on Graves Orbitopathy Guidelines for improvement of eye function and quality of life [4]; however, the mechanism by which selenium improves the manifestations of Graves ophthalmopathy remains unclear. Therefore, research into selenium may be a future trend.

### Rituximab

There has been substantial research into the effects of rituximab in Graves ophthalmopathy therapy, and many clinical trials have demonstrated that rituximab has therapeutic effects, with limited side effects [29]. As an anti-CD20 monoclonal antibody, rituximab can inhibit B cell activation and decrease inflammatory cytokine secretion [29]. As modern therapy strategies for Graves ophthalmopathy are concerned with individualization and precision, immune-based therapies have become increasingly popular, and numerous antibodies have been applied as new therapies. As one such antibody therapy method, rituximab has been researched in randomized controlled trials and fundamental research [29-32]. Additional research into this burst keyword will provide more detailed data in the coming years.

Research frontiers can be explored by identifying the most recent burst citations. As shown in Figure 9, the top five burst citations with the highest strength at the end of 2019 were as follows: (1) Bahn et al [21], which reviewed Graves ophthalmopathy knowledge and described the clinical and laboratory features of Graves ophthalmopathy, as well as its pathogenesis and therapeutic approaches; (2) Bartalena et al [4] listed current therapeutic recommendations for patients with different degrees of Graves ophthalmopathy; (3) Smith et al [1] described the clinical manifestations, pathogenesis, diagnosis, and therapy of GD, where Graves ophthalmopathy was considered an extrathyroid complication, the pathogenesis and manifestations of which are established; (4) Salvi et al [32] reported a randomized controlled trial on the effects of rituximab as therapy for active moderate to severe Graves ophthalmopathy, which demonstrated that rituximab led to better outcomes than intravenous methylprednisolone; and (5) Marcocci et al [33] demonstrated that selenium significantly improved outcomes in patients with Graves ophthalmopathy, compared with placebo in a randomized controlled trial, illustrating the importance of selenium supplementation for patients with Graves ophthalmopathy.

With the increasing amount of literature, visualization of data has become increasingly important and difficult for researchers to understand the current research progress from a large perspective [8]. As a tool software, CiteSpace explores future development trends and grasps the knowledge network map of Graves ophthalmopathy as a whole, rather than just aiming at a special topic such as typical reviews [13]. This study provides a reference for other researchers to understand the current research status and possible future directions in the field of Graves ophthalmopathy. The trend of further increase in the number of studies would attract more attention from researchers and further promote the development of this field. The analysis of references pointed out useful classical literature that could be regarded as the research cornerstone in this field and may help researchers understand the field of Graves ophthalmopathy faster and better. The analysis of the burst keyword indicated a possible future development direction in this field. Proliferation, selenium, and rituximab may represent the development direction of the mechanism and treatment in the field of Graves ophthalmopathy, respectively. Co-operation among authors, institutions, and countries would provide a reference for the introduction of academic resources, the development of co-operation, and the evaluation of academic achievements [13]. In addition, bibliometric and visual analysis and the use of CiteSpace were not only limited to the research in the field of Graves ophthalmopathy but also could be used in other fields because every discipline has its unique knowledge network system.

To our knowledge, this is the first bibliometric analysis of Graves ophthalmopathy using publication outputs from the Web of Science from 1999 to 2019. This study has some limitations. Although we attempted to collect all reports on Graves ophthalmopathy over the last 20 years, some were not included, such as gray literature and related reports in databases other than WoSCC. Furthermore, some recent articles will likely accrue more citations in the future, which may have led to the exclusion of high-quality studies from this analysis. Moreover, the number of citations does not fully represent the importance of an article, as some important articles may have few citations and self-citations can cause bias.

In conclusion, this bibliometric analysis highlights possible emerging trends in Graves ophthalmopathy research through analysis of publication outputs from 1999 to 2019, as well as the contributions of countries, institutions, authors, and journals. These results may inform clinical decision-making and future research.

## Appendix

Multimedia Appendix 1 Researching strategies and results.

## Abbreviations

GD: Graves disease
H-index: Hirsch index
IF: impact factor
WoSCC: Web of Science Core Collection Database
